# A 3D Microfluidic ELISA for the Detection of Severe Dengue: Sensitivity Improvement and Vroman Effect Amelioration by EDC–NHS Surface Modification

**DOI:** 10.3390/mi12121503

**Published:** 2021-11-30

**Authors:** Hinata Maeno, Pooi-Fong Wong, Sazaly AbuBakar, Ming Yang, Sing-Sin Sam, Juraina Jamil-Abd, Anusha Shunmugarajoo, Mahiran Mustafa, Rosaida Md Said, Eashwary Mageswaren, Azureen Azmel, Anilawati Mat Jelani

**Affiliations:** 1 Department of System Design, Tokyo Metropolitan University, Tokyo 191-0065, Japan; maeno-hinata@ed.tmu.ac.jp; 2 Department of Pharmacology, Faculty of Medicine, University of Malaya, Kuala Lumpur 50603, Malaysia; wongpf@um.edu.my; 3 Tropical Infectious Diseases Research and Educational Centre (TIDREC), University of Malaya, Kuala Lumpur 50603, Malaysia; sazaly@um.edu.my (S.A.); singsin@um.edu.my (S.-S.S.); juraina@um.edu.my (J.J.-A.); 4 WHO Collaborating Centre for Arbovirus Reference and Research (Dengue and Severe Dengue) MAA-12, University of Malaya, Kuala Lumpur 50603, Malaysia; 5 Medical Department, Tengku Ampuan Rahimah Hospital, Klang 41200, Malaysia; anukts3@gmail.com (A.S.); dresa712@hotmail.com (E.M.); aazmel@gmail.com (A.A.); 6 Medical Department, Raja Perempuan Zainab II Hospital, Kota Bharu 15200, Malaysia; mahiranmustafa@hotmail.com (M.M.); anilanazri@gmail.com (A.M.J.); 7 Medical Department, Ampang Hospital, Ampang 68000, Malaysia; drrosaida@moh.gov.my

**Keywords:** ELISA, microfluidics, EDC–NHS coupling

## Abstract

Serum is commonly used as a specimen in immunoassays but the presence of heterophilic antibodies can potentially interfere with the test results. Previously, we have developed a microfluidic device called: 3D Stack for enzyme-linked immunosorbent assay (ELISA). However, its evaluation was limited to detection from a single protein solution. Here, we investigated the sensitivity of the 3D Stack in detecting a severe dengue biomarker—soluble CD163 (sCD163)—within the serum matrix. To determine potential interactions with serum matrix, a spike-and-recovery assay was performed, using 3D Stacks with and without surface modification by an EDC–NHS (N-ethyl-N′-(3-(dimethylamino)propyl)carbodiimide/N-hydroxysuccinimide) coupling. Without surface modification, a reduced analyte recovery in proportion to serum concentration was observed because of the Vroman effect, which resulted in competitive displacement of coated capture antibodies by serum proteins with stronger binding affinities. However, EDC–NHS coupling prevented antibody desorption and improved the sensitivity. Subsequent comparison of sCD163 detection using a 3D Stack with EDC–NHS coupling and conventional ELISA in dengue patients’ sera revealed a high correlation (R = 0.9298, *p* < 0.0001) between the two detection platforms. Bland–Altman analysis further revealed insignificant systematic error between the mean differences of the two methods. These data suggest the potentials of the 3D Stack for further development as a detection platform.

## 1. Introduction

Enzyme-linked immunosorbent assay (ELISA) is one of the most commonly used methods in medical diagnostics, food analysis, and drug screening [1,2,3,4]. At present, most ELISAs are performed in 96-well polystyrene plates, whereby primary antibodies are immobilized onto its surface to detect specific antigen from a complex mixture by the proteins [1,2,3,4,5]. Although this method has high specificity, sensitivity, and the ability to perform multiple assays at a time, numerous limitations still exist. For example, antigen–antibody binding rate can be slow because antigen diffusion is dependent on Brownian motion [4,6].

Microfluidic devices enable a shorter turnaround time and improve the sensitivity of ELISA by shortening the diffusion distance of molecules and increasing the specific surface area [7]. Although a variety of microfluidic devices—such as channel patterning [8], microbeads-based devices [9], centrifuge discs [10], and paper-based [11] devices—have been developed, most laboratories still use the conventional 96-well plate ELISA method [12]. This is due to the high cost of device fabrication [13] and the need for peripheral equipment such as pumps and detectors [12]. In medical and biological applications, disposable devices are desirable to avoid biological contamination and false-positive signals; therefore, low-cost and mass-producible microfluidic devices are required [14]. However, the soft lithography technique, the most common microfluidics fabrication process, is costly because it requires photolithography for mold fabrication [13]. Even if the manufacturing cost of the device can be reduced, the conventional plate reader cannot be used, and a detector needs to be developed for quantification.

**Figure 1:**
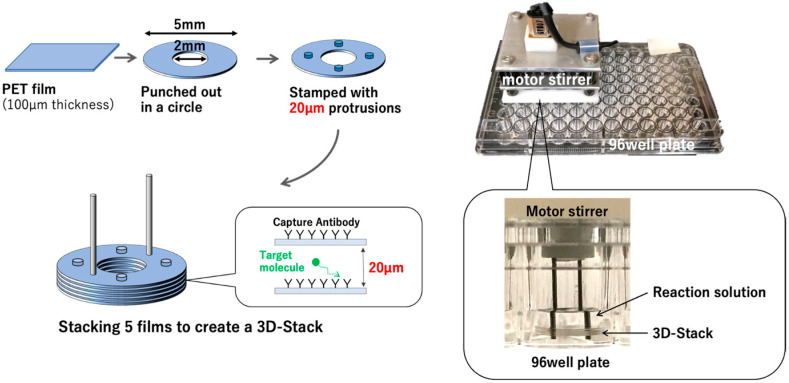
The fabrication and assembly of a 3D Stack. Briefly, PET films were punched out and stamped with 20 μm protrusions. The 3D Stack was assembled by stacking five PET films together. A motor stirrer was used to rotate the 3D Stack within the well.

To address these concerns, we have developed a low-cost microfluidic channel called 3D Stack that can be used in combination with 96-well plates (Figure 1). The 3D Stack is made by stacking polyethylene terephthalate (PET) films, with 20 µm gaps in between the films, to serve as microchannels to increase binding surface area. The entire assembly process of 3D Stack is performed using a press machine, which facilitates mass production and thereby lowers the cost of the device production. The entire 3D Stack is coated with capture antibodies and rotated in a 96-well plate to provide circulating flow within the microchannels to increase antigen binding. For subsequent reaction steps, the 3D Stack can be easily moved to the adjacent well containing the reagents; finally, to quantify the enzyme reaction, the 3D Stack is removed and the remaining reaction solution in the wells can be read using a conventional plate reader. The 3D Stack prototype was able to detect Rubella virus antigen with 1.9 times higher sensitivity than the conventional ELISA, using 96-well plates [15]. Suzuki et al. further modified the geometry of 3D Stack to enhance its sensitivity, and incorporated fluorescence detection resulting in two times higher detection fluorescence intensity than the conventional IgA ELISA [16,17]. Based on the promising results from these studies, we developed a 3D Stack ELISA for the detection of a severe dengue biomarker.

Thus far, the evaluation of the 3D Stack ELISA has been limited to the detection from a homogenous standard reagent which is a single protein solution. When using complex biological samples, such as serum, its matrix can potentially result in interference in immunoassay [18]. For example, rheumatoid factor (RF) and heterophilic antibodies (HA) can form complexes with specific ELISA antibodies and interfere with antibody–antigen binding [19]. Non-specific bindings with sample matrix can also interfere with antibody–antigen binding reactions. Holmberg et al. studied the adsorption behavior of albumin and IgG from human serum solutions on hydrophilic PET and found that albumin and IgG adsorption decreased in proportion to adsorption time and the serum concentration, due to competitive adsorption with serum protein [20,21,22]. This phenomenon is called the Vroman effect, which occurs because it is more thermodynamically stable in nature when high molecular weight proteins replace low molecular weight proteins [23,24]. The Vroman effect is also more likely to occur on hydrophilic surfaces, but 96-well microplates used in ELISA are generally hydrophobic [20]. Therefore, the Vroman effect is not an issue in a conventional ELISA using 96-well microplates. However, in microfluidic devices, hydrophilic surfaces are often used to reduce flow resistance [25,26]; therefore, they could be affected by the Vroman effect.

The contamination of substrates caused by non-specific adsorption of proteins, such as the Vroman effect, can be suppressed by self-assembling monolayers (SAM) [27,28,29]. Zhou et al. suppressed the nonspecific adsorption of proteins by modifying the poly(dimethylsiloxane) (PDMS) surface with poly(ethylene glycol) (PEG) chains [30]. The alkanethiol SAM is the most widely used for preventing nonspecific adsorption on gold surfaces [27]. Although, the SAM formation requires a high density of functional groups, which limits the number of substrates that can be used (PDMS, gold, glass, etc.). In addition, the reproducibility is low since the SAM is very sensitive to reaction conditions [31]. For microfluidic devices that are made using substrates which are difficult to make SAMs, such as the PET film used in the 3D Stack, there is still insufficient knowledge on how to prevent the Vroman effects of proteins in serum. In this study, we evaluated whether the Vroman effect can be prevented by covalently immobilize antibodies directly to a hydrophilic PET film.

Dengue is a mosquito-borne disease caused by dengue viruses and is endemic in many tropical and subtropical countries [32]. It can present as the mild self-limiting dengue fever, with or without warning signs, which can progress to the more serious forms, with severe plasma leakage and/or severe bleeding and/or multiple organ failures. Severe dengue can be fatal if patients are not properly treated [33]. Dengue fever is diagnosed by the detection of dengue non-structural protein 1 (NS1) or dengue-specific IgM and IgG levels, using ELISA or immunochromatographic (ICT)-based rapid detection test. Molecular tests, involving real-time reverse transcription-polymerase chain reaction (RT-PCR) [33,34] and RT-loop-mediated isothermal amplification (RT-LAMP) are also used for virus detection and genotyping [35]. The ELISA format test is a highly sensitive and specific, yet inexpensive, quantitative assay, which has remained as an important platform for the development of diagnostic tests. Although various diagnostic tests for dengue fever are commercially available, to date, there is no specific diagnostic test for severe dengue, owing to the lack of specific severe dengue biomarkers. Therefore, the diagnosis of severe dengue remains highly dependent on clinical assessment and non-dengue-specific laboratory parameters, such as platelets counts and hematocrit levels. Ab-Rahman et al. previously showed that differential expression levels of the soluble form of CD163 (sCD163), a scavenger receptor expressed on macrophages, can distinguish severe dengue patients from those with dengue fever [36]. Hence, sCD163 was selected as a target antigen for the present study. Here, we sought to determine whether serum matrix will interfere with the performance of 3D Stack ELISA and optimize the 3D Stack’s characteristics, such as geometry, rotating condition, and surface properties, to further improve its detection sensitivity. Finally, we evaluated the performance of the 3D Stack ELISA for sCD163 detection using dengue patients’ sera.

## 2. Materials and Methods

### 2.1. Materials and Instruments

Human CD163 ELISA kits (DY1607) were purchased from R&D systems. Sodium hydroxide (NaOH), 2-(N-morpholino)ethanesulfonic acid (MES), N-(3-Dimethylaminopropyl)-N′-ethylcarbodiimide hydrochloride (EDC), and N-Hydroxysuccinimide (NHS) were acquired from Sigma-Aldrich. Phosphate-buffered saline (PBS) and 0.2 µm asymmetrical polyethersulfone (aPES) membrane filter were purchased from Thermo Fisher Scientific. TMB 2-Component Microwell Peroxidase Substrate Kit and TMB stop solution were purchased from SeraCare Life Sciences Inc. Polyclonal rabbit anti-mouse immunoglobulins/HRP (P026002-2) was purchased from Agilent Technologies, Inc. Deionized water was used in all assays. All chemicals and reagents were of analytical grade and used without further purification. The 3D Stacks were fabricated by Polyester Film Lumirror™ (Toray Industries, Inc., Tokyo, Japan). The detailed fabrication method was described in a previous study [17]. The absorbance was measured by Microplate reader Sunrise™ (Tecan Group Ltd., Mannendorf, Switzerland). Particle size distribution, by dynamic light scattering (DLS) technology, was determined using Zetasizer Nano (Malvern Panalytical, Malvern, UK).

### 2.2. Recruitment of Patients and Study Approval

A total of twenty-four dengue patients’ sera were used in the present study. Patients were clinically and laboratory confirmed for dengue. Laboratory confirmation of dengue was based on the detection of dengue-specific IgM antibody or dengue non-structural protein 1 (NS1) antigen or dengue-specific IgG antibody in the respective hospital. Dengue case classification was performed based on the 2009 World Health Organization (WHO) revised dengue classification criteria by clinicians of the respective hospital. This study was approved by the Medical Research and Ethics Committee of the Ministry of Health, Malaysia (Ethics No. NMRR-16-2814-29003) and conformed to the Declaration of Helsinki and Malaysian Good Clinical Practice (GCP) guidelines. Blood was collected in serum separator tube (SST) and spun at 1200× *g* for 10 min. Sera were aliquoted into new tubes and kept in −20 °C until further study.

### 2.3. Hydrolysis of 3D Stack

Prior to the immunoassay, the 3D Stack surfaces were hydrolyzed with NaOH to make the surfaces hydrophilic. By hydrolyzing the PET films of the 3D Stack with NaOH, carboxyl and hydroxy groups are formed on the 3D Stack surface, which improved the hydrophilicity of the flow channel [37]. The 3D Stacks were submerged in 2.5 M NaOH and incubated at 50 °C for 2 h in a water bath. Following hydrolysis, the 3D Stacks were removed from the NaOH aqueous solution and rinsed with deionized water. The hydrolyzed 3D Stacks were used immediately for ELISA. The scheme diagram is in Supplementary Information.

### 2.4. Sandwich ELISA Using 3D Stack and Conventional Method

**Figure 2:**
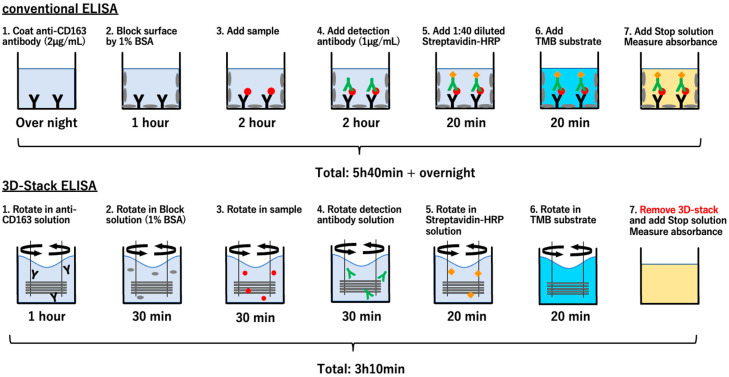
Scheme of sandwich ELISA using conventional 96-well plate and 3D Stack methods. Conventional ELISA for sCD163 was performed according to the protocols provided by the manufacturer with similar reagent concentrations used for both methods.

For ELISA using the 3D Stack, mouse anti-human CD163 antibody was diluted to 2 µg/mL with 0.1 M PBS and added to a 96-well plate at 100 µL per well. One 3D Stack was inserted to each well and rotated by a motor stirrer at 2000 rpm for 1 h at room temperature. After incubation, the 3D Stack was transferred to the well, containing 100 µL of washing solution (1% Tween 20 in 0.1 M PBS), and rotated at 2000 rpm for 10 s, 5 times. For blocking, 100 µL of 1% BSA in PBS was added to a new well. The 3D Stack was inserted and rotated for 30 min at room temperature. After blocking, sera were diluted at 1:100 dilution with 1% BSA in PBS and added to each new well. The 3D Stack was inserted into the well and rotated at 2000 rpm for 30 min, followed by washing. Next, 100 µL of detection antibody (biotinylated anti-CD163), diluted to 1 µg/mL with 1% BSA in PBS, was added to each new well. The 3D Stack was inserted and rotated at 2000 rpm for 30 min at room temperature, followed by washing. Then, 100 µL of streptavidin-HRP, diluted at 1:40 with 1% BSA in PBS was added to each new well. The 3D Stack was inserted and rotated at 2000 rpm for 20 min at room temperature in the dark, followed by washing. Lastly, 100 µL of TMB substrate was added into each new well. The 3D Stack was inserted and rotated at 2000 rpm for 20 min at room temperature in the dark followed by washing. After the reaction, the 3D Stack was removed from the well and the absorbance of the remaining solution in the well was measured at 450 nm wavelength, with a reference wavelength of 570 nm, using a microplate reader. For conventional ELISA using only 96-well plates, the same concentrations of reagents were used, and the assay was performed according to the protocol provided by the manufacturer (Human CD163 ELISA kit, catalog no. DY1607, R&D Systems). Briefly, the incubation times were as follows: overnight for capture antibody, 1 h for blocking, 2 h for sample, 2 h for detection antibody, 20 min for streptavidin-HRP, and 20 min for TMB substrate. Corrected absorbance values were obtained after subtracting absorbance of blank wells. Scheme of sandwich ELISA using conventional 96-well plate and 3D Stack methods is shown in Figure 2.

### 2.5. Spike-and-Recovery Assay

To determine if serum matrices interfere with ELISA, recombinant human CD163 (5 ng/mL) was spiked into sera diluted at 0.5, 1.0, and 10% with 1% BSA in PBS, and the levels of spiked CD163 from sera were detected using 3D Stack or conventional ELISA. The absorbance values were compared to one of the standard samples, having the same CD163 concentration. In the absence of interference from serum matrices, both standard and spiked samples should show identical absorbance (100% recovery). As human serum has a certain CD163 level even in a healthy state, unspiked serum was also tested and its absorbance value was subtracted from those of the spiked samples. In this experiment, we used serum samples from healthy adult volunteers.

### 2.6. Detection of the Remaining Coated Antibodies on the Surface of 3D Stack

To quantify the amount of coated mouse anti-CD163 antibodies remaining on the 3D Stack surface, polyclonal rabbit anti-mouse immunoglobulins/HRP was used. Following the incubation of 2 µg/mL of anti-CD163 antibody with diluted sera (0.5, 1.0, and 10%), the 3D Stack was rotated in 100 µL of wash buffer (1% Tween 20 in 0.1 M PBS) at 2000 rpm, 5 times. Following washing, polyclonal rabbit anti-mouse Immunoglobulins/HRP was diluted to 1:1000 with 0.1 M PBS, and 100 µL was added to the new well. A 3D Stack was inserted into each well and rotated for 30 min at 2000 rpm in the dark. After incubation, 100 µL of TMB was added into a new well. The 3D Stack was inserted to the well and rotated at 2000 rpm for 10 min in the dark, followed by washing. After the reaction, the 3D Stack was removed from the well and the absorbance of the remaining solution in the well was measured at 450 nm wavelength, with a reference wavelength of 570 nm, using a microplate reader.

### 2.7. EDC–NHS Coupling of Antibody

**Figure 3:**
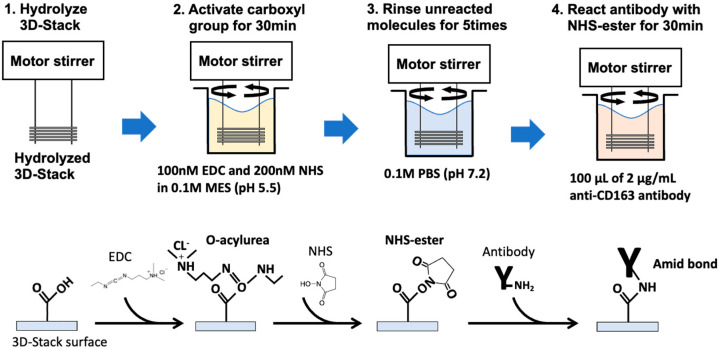
Scheme of EDC–NHS coupling.

To covalently couple the primary amine of the anti-CD163 antibody to the carboxyl group on the hydrolysed 3D Stack surface, EDC–NHS coupling was performed. First, the hydrolysed 3D Stack was rotated in 0.1 M MES buffer (pH 5.5) containing 100 nM EDC and 200 nM NHS for 30 min to form an amine-reactive NHS-ester from the carboxyl groups on the surface. Next, the reacted 3D Stack was rotated in 0.1 M PBS (pH 7.2) for 10 s, repeated 5 times, to remove unreacted molecules. Lastly, 100 µL of 2 µg/mL anti-CD163 antibody in 0.1 M PBS (pH 7.2) was added into a new well and the 3D Stack was rotated for 30 min at 2000 rpm to facilitate the reaction of NHS-ester on the 3D Stack surface with the primary amine of the anti-CD163 antibody. Scheme of EDC–NHS coupling is shown in Figure 3.

### 2.8. Statistical Analysis

An unpaired *t*-test was performed for comparison between two groups of data. Simple linear regression analysis was used to evaluate the correlation between 3D Stack and 96-well ELISA. A Bland–Altman analysis was performed to assess the degree of agreement between the two quantitative methods. A Shapiro–Wilk test was performed to determine if the mean differences are normally distributed. All statistical analyses were performed using Graph Pad Prism version 9.0.2. A *p*-value lower than 0.05 was considered significant.

## 3. Results

### 3.1. Detection Sensitivity of 3D Stack from Standard Sample

**Figure 4:**
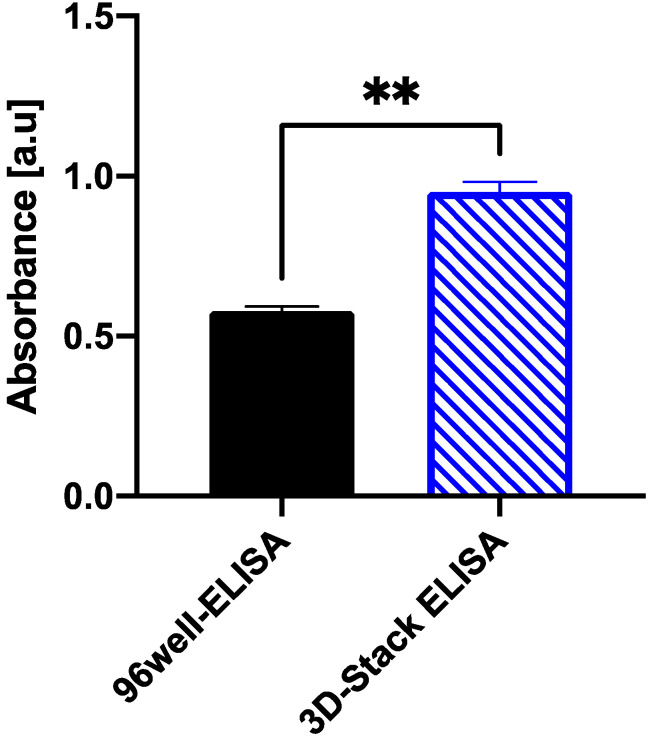
Comparison of absorbance between 3D Stack ELISA and conventional 96-well ELISA. Both methods were used to detect 5 ng/mL sCD163 standard solution and the corrected absorbance values were compared. Statistical analysis was performed using unpaired *t*-test. *p* < 0.05 is considered as statistically significant, ** *p* < 0.01; a.u.—arbitrary unit.

To confirm the detection sensitivity of 3D Stack ELISA, human CD163 standard samples were measured, and the absorbance was compared with those measured using the conventional 96-well plate sandwich ELISA method. Absorbance levels captured by 3D Stack ELISA were higher than those of the conventional 96-well ELISA (*p* < 0.01) (Figure 4). In addition, the total turnaround time of the conventional 96-well ELISA was 5 h 40 min (overnight), compared with 3 h 10 min for 3D Stack ELISA. This suggests that 3D Stack ELISA was not only more sensitive than the 96-well ELISA but also requires a shorter incubation time when used for detection in a single protein solution.

### 3.2. Detection from Serum Samples

**Figure 5:**
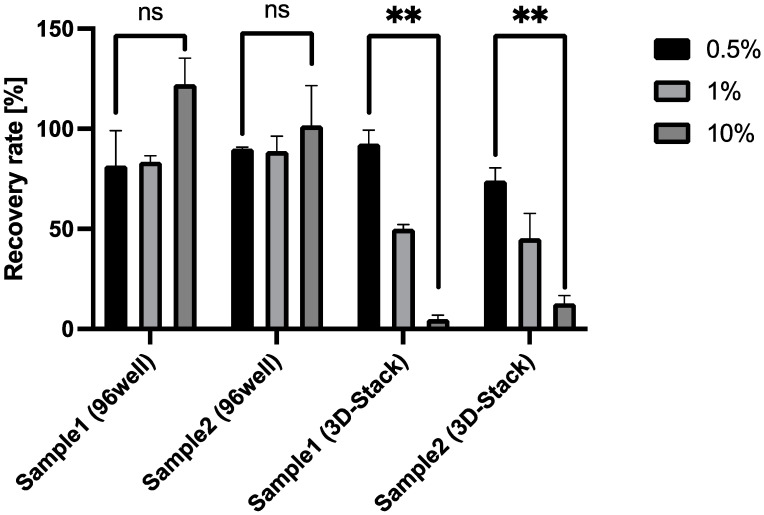
Recovery of spiked sCD163 by 96-well or 3D Stack ELISA. A measure of 5 ng/mL CD163 were spiked into 2 serum samples at 3 dilutions (0.5, 1.0, and 10%) and the recovery rates of conventional 96-well ELISA (2 groups on the left) or 3D Stack ELISA (2 groups on the right) were determined. Statistical analysis was performed using an unpaired *t*-test, whereby *p* < 0.05 was considered as statistically significant. ** *p* < 0.01; a.u.—arbitrary unit.

A spike-and-recovery assay was conducted to determine the effect of serum matrix on the sensitivity of 3D Stack ELISA. Figure 5 and Table 1 show the recovery rate of sCD163 from diluted serum samples (0.5–10% dilution), spiked with 5 ng/mL sCD163, by 3D Stack and conventional 96-well ELISA. The recovery rate of sCD163 by 3D Stack decreased in proportion to serum concentration, with the lowest recovery rate at the highest serum dilution (*p* < 0.01). In contrast, the recovery rates remained consistent (80–120%) for all serum dilutions using conventional 96-well ELISA (Figure 5 and Table 1), suggesting that the Vroman effect did not occur, as 96-well plates are hydrophobic and not hydrophilic, making them permissible to the Vroman effect. However, the recovery rates were comparable for both methods for diluted sera at 0.5%, suggesting that low serum matrix concentration did not significantly affect the recovery of the 3D Stack. Taken together, this result indicates that both serum samples did not contain specific interfering substances for antigen–antibody interactions, but the characteristics of the 3D Stack, such as microflow path, rotation, and surface properties, might have contributed to the interference effect.

### 3.3. The Effect of 3D Stack Characteristics on the Detection of sCD163 from Serum

**Figure 6:**
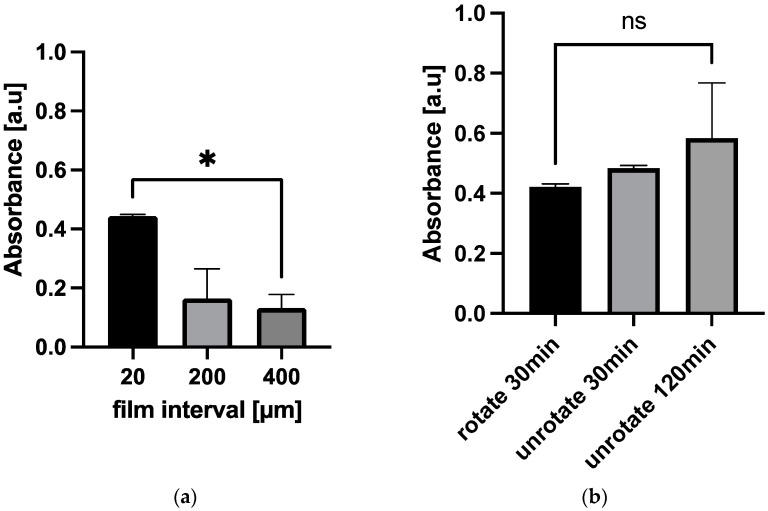
Effects of film interval and rotation on absorbance. (**a**) Comparison of absorbance between different film gap 3D Stack. * *p* < 0.1. (**b**) Comparison of absorbance between different rotate conditions. Statistical analysis was performed using unpaired *t*-test. *p* < 0.05 was considered as statistically significant; a.u.—arbitrary unit; ns—not significant.

To investigate the causes of the lower antigen recovery rate of the 3D Stack, the 3D Stack’s characteristics were changed. First, the width of the microflow paths (film gap of 3D Stack) were increased from 20 µm to 400 µm and then used to detect 1% diluted serum spiked with 5 ng/mL sCD163. The absorbance readings were reduced in proportion to the film gap (*p* > 0.05), suggesting that the small flow gap was not the main cause of the low antigen recovery rate (Figure 6a). The reduction in recovery rate in proportion to the film gap could be contributed by flow velocity. The fluidic flow within the 3D Stack is accelerated by centrifuge force. However, the increased film gap resulted in decreased flow velocity, which then reduced the reaction’s efficiency. Next, the rotation time was changed, whereby the 3D Stacks were either left static for 30 or 120 min or rotated for 30 min during sample incubation, to eliminate shear force caused by rotation. However, the absorbance readings remained unchanged (*p* = 0.3386) even in the 120 min static condition (Figure 6b). These results suggest that the microflow path of the 3D Stack and shear force did not contribute to lower recovery rate; however, other factors, such as the surface property of the 3D Stack, may play a role.

### 3.4. The Influence of Vroman Effect on the Detection of sCD163 from Serum

**Figure 7:**
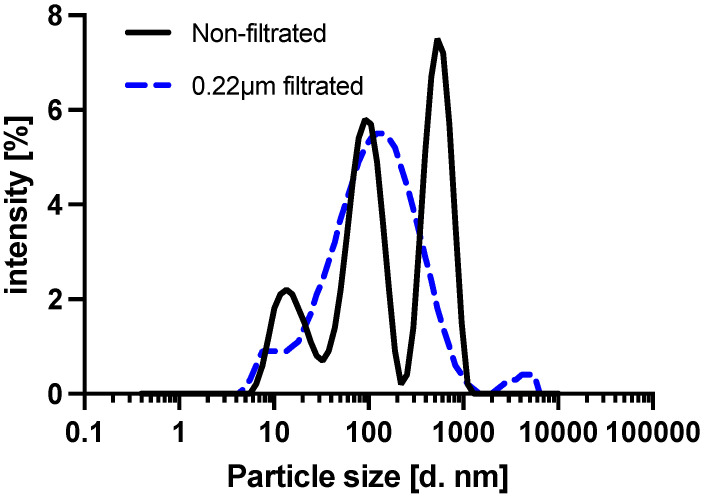
Particle diameter distribution of non-filtered and filtered serum. Serum was diluted at a 1:10 dilution with PBS and particle size; d was measured by dynamic light scattering (DLS) technique using a Zetasizer (brand) and wavelength, etc. Measurement duration was 60 s.

**Figure 8:**
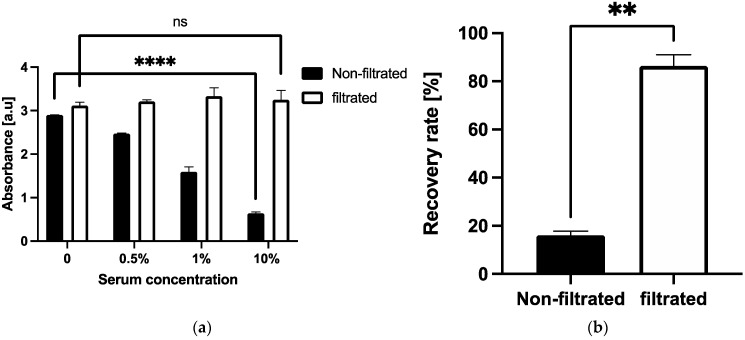
sCD163 detection in non-filtered and filtered sera at various dilutions. (**a**) CD163 levels in diluted non-filtered and filtered sera. (**b**) CD163 recovery rate from non-filtered and filtered serum using 3D Stack ELISA. Statistical analysis was performed using unpaired *t*-test, whereby *p* < 0.05 is considered as statistically significant. ** *p* < 0.01; **** *p* < 0.0001; a.u.—arbitrary unit; ns—not significant.

The Vroman effect is observed when low molecular weight proteins which are reversibly adsorbed onto the surface are displaced by high molecular weight proteins with higher absorption affinities [23,38]. Hydrophilic surfaces also produce a higher chance of protein displacement [39]. As the 3D Stack surface is hydrophilic, we hypothesized that the anti-CD163 antibody initially adsorbed on the 3D Stack surface was displaced by the serum proteins. Particle size distribution of serum was first measured by dynamic light scattering (DLS) technique to determine the distribution of proteins with various sizes in serum. There were 3 major peaks that were observed at 10, 100, and 500 nm (Figure 7, solid line). The 100 and 500 nm peaks indicate serum contains larger proteins than the anti-CD163 antibody, since the diameter of IgG, alike to the anti-CD163 antibody, is approximately 10–15 nm [40]. Large proteins were hence removed, by filtering the serum with a 0.22 µm asymmetrical polyethersulfone (aPES) membrane filter. Serum filtration resulted in one major peak of approximately 100 nm (Figure 7, dotted line). To confirm whether antibody displacement by high molecular weight proteins occurred in the present study, we compared sCD163 detection in non-filtered and filtered sera. In non-filtered serum, the absorbance readings decreased in proportion to the incubated serum concentration (*p* < 0.0001). However, for filtered serum, there was no change in absorbance readings (Figure 8a). These results suggest that anti-CD163 antibodies on the 3D Stack surface were displaced by large serum proteins. To further validate this, a spike-and-recovery assay was conducted with the non-filtered and filtered sera to investigate the effect of high molecular weight serum protein on 3D Stack ELISA. Figure 8b shows that the recovery rate has significantly improved by serum filtration (*p* = 0.0026). Therefore, it can be concluded that the serum interference on 3D Stack ELISA was caused by the anti-CD163 antibody displacement with large serum proteins.

### 3.5. Antibody Immobilization by EDC–NHS Coupling

**Figure 9:**
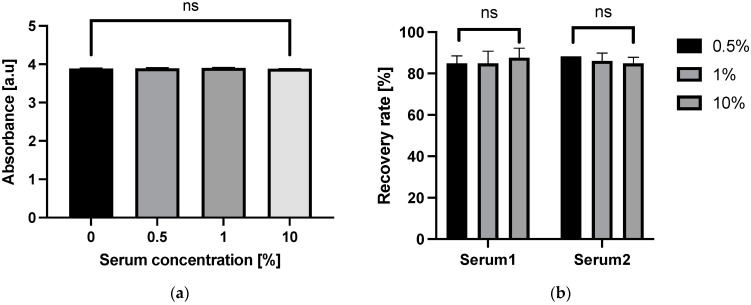
Effect of EDC–NHS coupling. (**a**) sCD163 levels detected in diluted sera following EDC–NHS coupling of 3D Stack surface. (**b**) sCD163 recovery rate from sera using 3D Stack ELISA with EDC–NHS coupling. Statistical analysis was performed using unpaired *t*-test, whereby *p* < 0.05 was considered as statistically significant. a.u.—arbitrary unit; ns—not significant.

To prevent the Vroman effect, the anti-CD163 antibody was covalently immobilized onto the 3D Stack surface by EDC–NHS coupling. Following EDC–NHS coupling, we measured the amounts of antibodies remaining on the surface after serum incubation using an anti-mouse IgG antibody and found no decrease in the absorbance readings, even in the highest dilution (10% serum, Figure 9a). In addition, the spike-and-recovery assay showed the recovery rate was over 80% for all serum concentrations (Figure 9b). From these results, we confirmed that covalent immobilization by EDC–NHS coupling prevented the anti-CD163 antibody displacement and overcame the serum interference on 3D Stack ELISA.

### 3.6. Detection of sCD163 from Dengue Patients’ Sera

**Figure 10:**
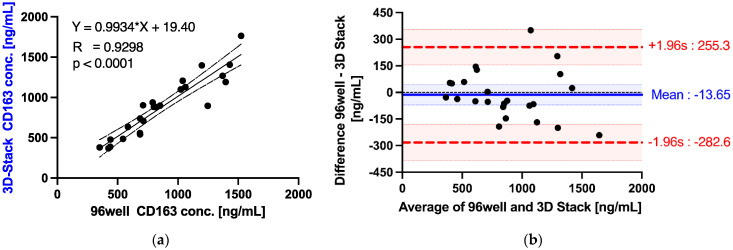
Correlation and limits of agreement between 3D Stack and 96-well ELISA methods. (**a**) Correlation analysis of sCD136 concentrations, detected by conventional 96-well ELISA and 3D Stack ELISA. (**b**) Bland–Altman plot. Horizontal lines are drawn at the mean difference (blue) and at the limits of agreement (red). The shaded areas represent confidence interval limits for mean and agreement limits. Simple linear regression and Bland–Altman analysis was performed using Graph Pad Prism version 9.0.2.

The performance of the 3D Stack ELISA with EDC–NHS coupling for sCD163 detection was further evaluated using dengue fever patients’ sera. A total of 24 dengue-confirmed patients’ sera were tested and compared with results obtained with a commercially available 96-well plate ELISA kit for sCD163. Before correlation test and Bland–Altman analysis, Shapiro–Wilk test was performed to confirm the normality of the differences using 96-well or 3D Stack. Data normality was verified, as shown in Table 2. Figure 10a shows the correlation plot of sCD163 concentrations between 3D Stack and 96-well ELISA. The correlation coefficient R was 0.9298 (*p* < 0.0001), indicating that the 3D Stack ELISA method is highly correlated to the 96-well ELISA method. Bland–Altman analysis further revealed the mean difference between two methods was −13.65 ± 137.2 ng/mL and the limits of agreement were −282.6 and 255.3 ng/mL (Table 3 and Figure 10b). Furthermore, the line of equality was within the confidence interval of the mean difference, CI [−71.34, 44.05], indicating that the bias or systematic difference of 13.65 is insignificant (Figure 10b).

## 4. Discussion

In the present study, we found that large serum proteins (>500 nm) displaced coated antibodies on the 3D Stack’s surface, resulting in reduced sensitivity of the 3D Stack ELISA. This phenomenon is called the Vroman effect, which was introduced by Vroman and Adams [23]. The protein adsorption process is entropically driven, and the entropy gain comes from dehydration of the absorbent surface and structural rearrangements inside the protein molecule [41]. In particular, high molecular weight proteins have a weak internal cohesion, resulting in a large conformational entropy gain by adsorption [29]. Thus, protein adsorption is initially influenced by diffusion and small proteins cover the surface; however, over time, higher-affinity proteins can replace lower-affinity proteins in a dynamic process [42]. The Vroman effect is also more likely to occur on hydrophilic surfaces, but 96-well microplates used in ELISA are generally hydrophobic [20]. Therefore, the Vroman effect is not an issue in a conventional ELISA using 96-well microplates. In the spike-and-recovery assay, the recovery rate of conventional ELISA remained consistent (80–120%) for all serum dilutions. In the case of the 3D Stack ELISA, the surface was hydrophilized by hydrolysis of PET to reduce flow resistance. Additionally, the micro-flow channel shortened the diffusion distance, so that large proteins can reach the surface within a short reaction time. Therefore, the anti-CD163 antibodies, initially coated on the 3D Stack, seemed to have high chance of being displaced by large serum proteins.

To prevent the Vroman effect, the anti-CD163 antibody was covalently immobilized onto the 3D Stack surface by EDC–NHS coupling, and we confirmed that this treatment overcame serum interference on the 3D Stack ELISA. Sam et al., (2010) reported that selected concentrations of EDC and NHS affect the formation of unwanted surface by-products [43]. In addition, Tonigold et al., (2018) also found that antibodies preferentially bind via the Fc region to the carboxyl groups of nanoparticles, and the Fab region (antigen-binding region) was less exposed with EDC–NHS coupling [44]. The orientation and the amount of immobilized capture antibodies play a critical role in the performance of an immunoassay [45]. Therefore, our future study should include surface modification, considering not only antibody displacement, but also the orientation and the amount of capture antibodies, to improve the sensitivity of the 3D Stack ELISA.

Finally, we detected sCD163 from 24 dengue fever patients’ sera using 3D Stacks with EDC–NHS coupling and compared the sCD163 levels with conventional ELISA; results showed that the 3D Stack ELISA is highly correlated to conventional 96-well ELISA and could be applicable for clinical diagnosis. The magnitude of systematic difference or bias of 3D Stack was also insignificant. Bland–Altman analysis identified the agreement interval, within which, 95% of the differences of 3D Stack, compared with the 96-well ELISA method, fell. Nevertheless, whether this range is clinically acceptable requires further evaluation of more clinical samples. In addition, the overall short assay turnaround time (3 h 10 min) was approximately two times faster than the conventional 96-well plate ELISA, this could be a result of rotating the 3D Stack to increase binding efficiency. When channels reached the micron scale and the capture fraction of the bulk analyte became much higher, like the 3D Stack, the reaction became diffusion-limited [46]. Therefore, increasing rotation speed is presumed to shorten the reaction time of the 3D Stack ELISA, since the mass flux to the surface will increase with convection. Additionally, the surface treatment method and fabrication technologies of the 3D Stack offer future opportunities to be explored in the development of microfluidic devices and diagnosis.

## 5. Conclusions

The Vroman effect, as a result of the displacement of coated antibodies on the 3D Stack surface by large serum proteins (>500 nm), has reduced the sensitivity of the 3D Stack ELISA. The Vroman effect, however, can be prevented by using EDC–NHS coupling to covalently immobilize the anti-CD163 antibody onto the 3D Stack surface. In addition, we found that the sensitivity reduction by the Vroman effect was not caused by the geometry of the channel or the shear force, but by the surface characteristics of the hydrophilized PET. Therefore, when designing devices for ELISA using hydrophilic materials, this phenomenon should be taken into consideration and the capture antibody should be immobilized by covalent bonding. Finally, we demonstrated that 3D Stacks with EDC–NHS coupling could detect the biomarker of severe dengue, sCD163, with results comparable to that of conventional ELISA, without significant systematic error between the mean differences of the two methods. Collectively, these data show the strong potentials of the 3D Stack as a detection platform for the development of diagnostic kits.

## Figures and Tables

**Table 1:** Recovery of spiked sCD163 by 96-well or 3D Stack ELISA.

Serum Dilution [%]	Recovery Rate (% ±SD)			
	Sample1 (96-Well)	Sample2 (96-Well)	Sample1 (3D Stack)	Sample2 (3D Stack)
0.5	81.7 ± 17.3	90.0 ± 0.81	92.6 ± 6.73	74.2 ± 6.24
1	83.6 ± 3.00	88.8 ± 7.47	50.0 ± 2.14	45.3 ± 12.5
10	122 ± 13.0	101 ± 19.9	5.00 ± 2.00	12.8 ± 3.79

**Table 2:** Shapiro–Wilk test.

	96-Well Elisa	3D Stack	Difference
w	0.9398	0.9536	0.9621
*p* value	0.1616	0.3244	0.483
alpha	0.05	0.05	0.05
normality	yes	yes	yes

**Table 3:** Bland–Altman plot statistics.

Parameter	Unit	Standard Error Formula	Standard Error (*se*)	*t* Value for 24 Degrees of Freedom	Confidence (*se* * *t*)	Confidence Intervals	
						from	to
number (*n*)	24						
degrees of freedom (*n* − 1)	23						
$\mathrm{difference}\;\mathrm{mean}\;(\bar{d}$)	−13.65	$\sqrt{s^{2}/n}$	28.01	2.06	57.69	−71.34	44.05
standard deviation (*s*)	137.2						
$\bar{d}$ − 1.96*s*	−282.6	$\sqrt{3s^{2}/n}$	48.51	2.06	99.92	−382.5	−182.6
$\bar{d}$ + 1.96*s*	255.3	$\sqrt{3s^{2}/n}$	48.51	2.06	99.92	155.3	355.2

## Supplementary Materials

The following are available online at https://www.mdpi.com/2072-666X/12/12/1503/s1, Figure S1: Dimensions of the 3D-Stack and fabricated 3D-Stack. Figure S2: Observed image and cross-sectional profile of a protrusion. Figure S3: Image of 3D-Stack operation. Figure S4: Hydrolysis reaction of PET. Figure S5: Scheme of hydrolysis. Figure S6: Relationship between reaction time and contact angle when reacting with 2.5 M NaOH at 50 °C. The contact angle became the smallest at a reaction time of 120 min, after which there was almost no change. Therefore, the reaction time was set to 120 min. Figure S7: Analytical model. Figure S8: Effect of film gap on radial velocity. Video S1: 3D-Stack operation.
