# Supplementary material for: A 3D Microfluidic ELISA for the Detection of Severe Dengue: Sensitivity Improvement and Vroman Effect Amelioration by EDC–NHS Surface Modification
Source: Micromachines (Basel). 2021 Nov 30;12(12):1503. doi: 10.3390/mi12121503 (PMC8715748; doi:10.3390/mi12121503)
Supplement: Supplementary file 1 [file micromachines-12-01503-s001.zip › micromachines-1433425 - Support information for proofreading.pdf]

A 3D Microfluidic ELISA for the Detection of Severe Dengue: Sensitivity Improvement and Vroman Effect Amelioration by EDC–NHS Surface Modification

Hinata Maeno [1], Pooi-Fong Wong [2], Sazaly AbuBakar [3,4,5], Ming Yang [1,*], Sing-Sin Sam [3,4], Juraina Jamil-Abd [3,4], Anusha Shunmugarajoo [5], Mahiran Mustafa [6], Rosaida Md Said [7], Eashwary Mageswaren [5], Azureen Azmel [5] and Anilawati Mat Jelani [6]

1 Department of System Design, Tokyo Metropolitan University, Tokyo 191-0065, Japan; maeno-hinata@ed.tmu.ac.jp
2 Department of Pharmacology, Faculty of Medicine, University of Malaya, Kuala Lumpur 50603, Malaysia; wongpf@um.edu.my
3 Tropical Infectious Diseases Research and Educational Centre (TIDREC), University of Malaya, Kuala Lumpur 50603, Malaysia; sazaly@um.edu.my (S.A.); singsin@um.edu.my (S.-S.S.); juraina@um.edu.my (J.J.-A.)
4 WHO Collaborating Centre for Arbovirus Reference and Research (Dengue and Severe Dengue) MAA-12, University of Malaya, Kuala Lumpur 50603, Malaysia
5 Medical Department, Tengku Ampuan Rahimah Hospital, Klang 41200, Malaysia; anukts3@gmail.com (A.S.); dresa712@hotmail.com (E.M.); aazmel@gmail.com (A.A.)
6 Medical Department, Raja Perempuan Zainab II Hospital, Kota Bharu 15200, Malaysia; mahiranmustafa@hotmail.com (M.M.); anilanazri@gmail.com (A.M.J.)
7 Medical Department, Ampang Hospital, Ampang 68000, Malaysia; drrosaida@moh.gov.my
* Correspondence: yang@tmu.ac.jp

1. 3D-Stack Fabrication and Operation

The 3D-Stack is made by stacking 100μm thick polyethylene terephthalate (PET) films, with 20 μm gaps in between the films to serve as microchannels to increase binding surface area. Polyester Film Lumirror™ (Toray Industries, Inc.) was used for 3D-Stack in this study. The entire assembly process of 3D-Stack is performed using a press machine, which facilitates mass production and thereby, lowers the cost of the device production. The following figure shows the dimensions of the 3D-Stack and a photo of the fabricated 3D-Stack.

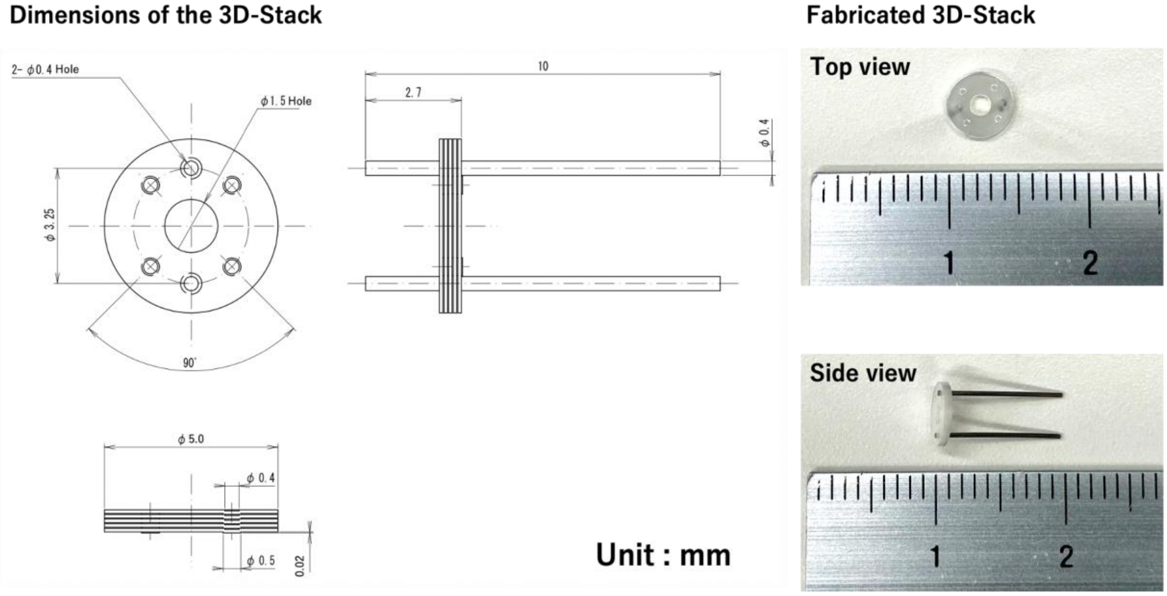

Figure S1. Dimensions of the 3D-Stack and fabricated 3D-Stack.

The four circular protrusions on the surface of the film to prevent the films from adhering to each other were checked using a confocal laser microscope (OLS500, Olympus)

to see if they were 20 μm in design dimension, and the height was 21.685 μm (Figure S2). This indicates that the gap between the films is sufficient to allow liquid to pass through.

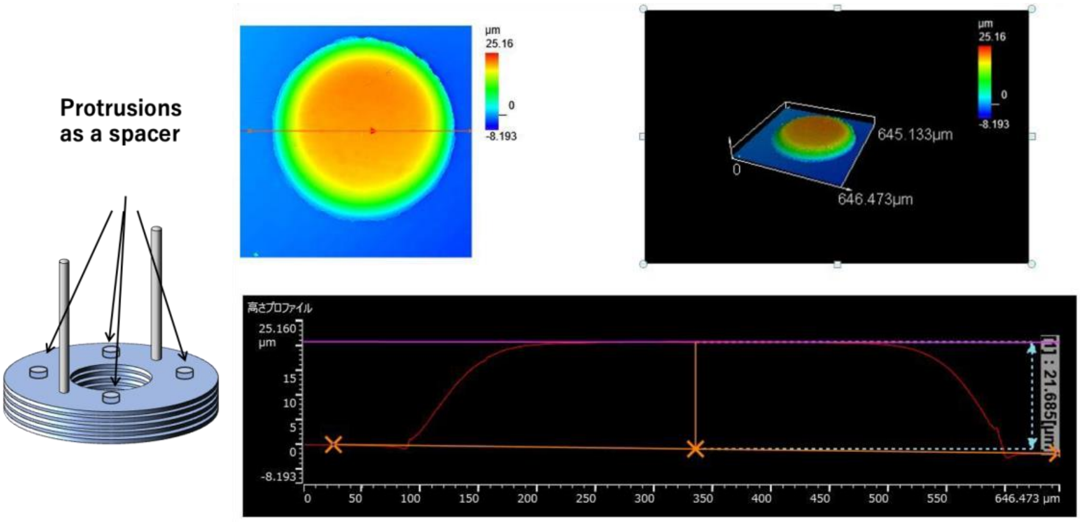

Figure S2. Observed image and cross-sectional profile of a protrusion.

The entire 3D-Stack is coated with capture-antibodies and rotated in a 96-well plate to provide circulating flow. For subsequent reaction steps, the 3D-Stack can be easily moved to the adjacent well containing the reagents and finally to quantify the enzyme reaction, the 3D-Stack is removed and the remaining reaction solution in the wells can be read using a conventional plate reader.

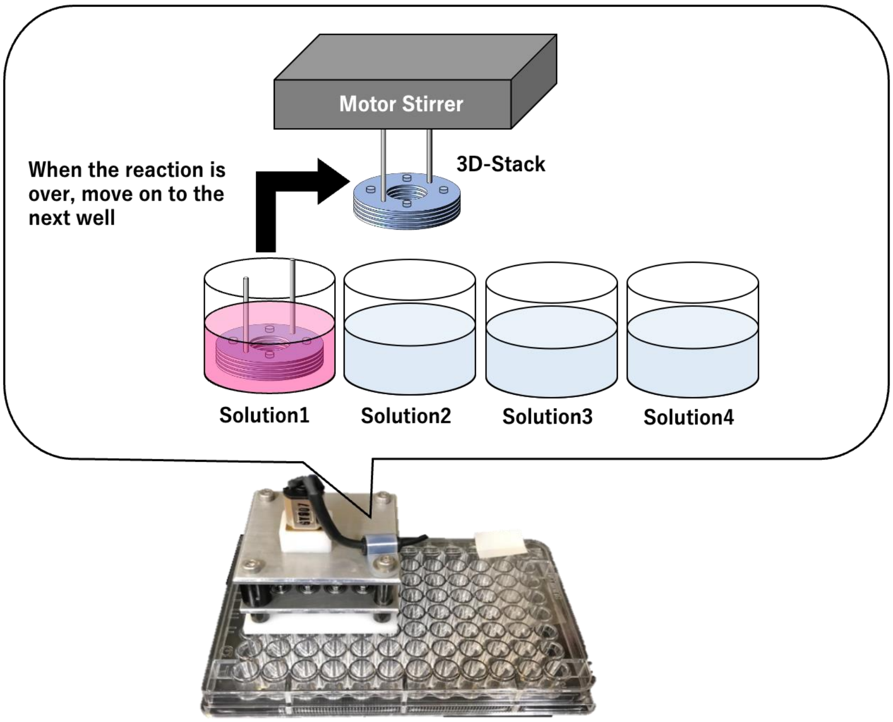

Figure S3. Image of 3D-Stack operation.

2. Scheme of Hydrolysis

Prior to the immunoassay, the 3D-Stack surfaces were hydrolysed with NaOH to make the surfaces hydrophilic. By hydrolyzing the PET films of the 3D-Stack with NaOH,

carboxyl and hydroxy groups are formed on the 3D-Stack surface, which improve the hydrophilicity of the flow channel[1] (Figure S4). 3D-Stacks were submerged in 2.5 M NaOH and incubated at 50°C for 2h in a water bath. Following hydrolysis, the 3D-Stacks were re-moved from the NaOH aqueous solution and rinsed with deionized water (Figure S5). The reaction conditions (concentration and temperature) were based on Atthoffs's research [2], and the reaction time was optimized by experiment (Figure S6).

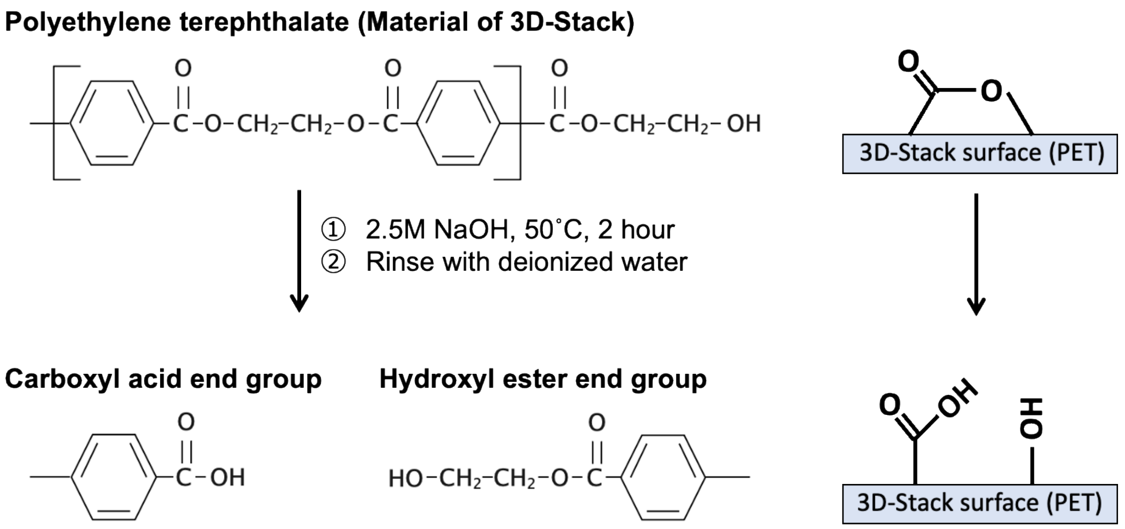

Figure S4. Hydrolysis reaction of PET.

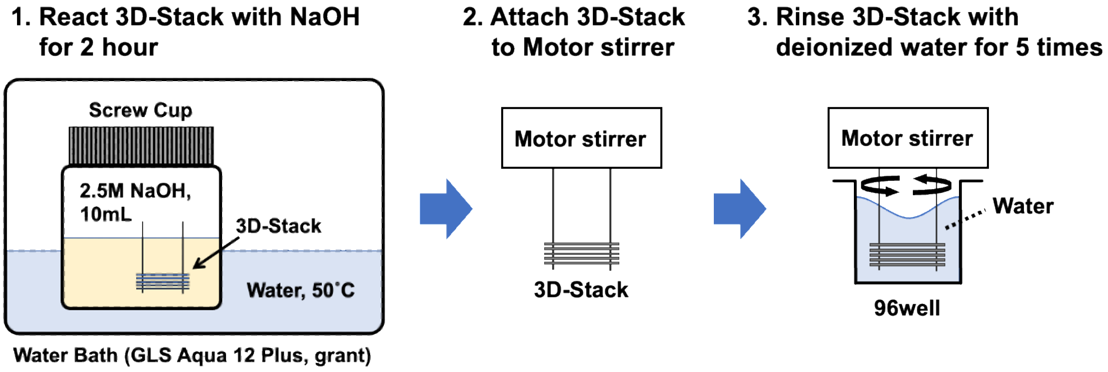

Figure S5. Scheme of hydrolysis.

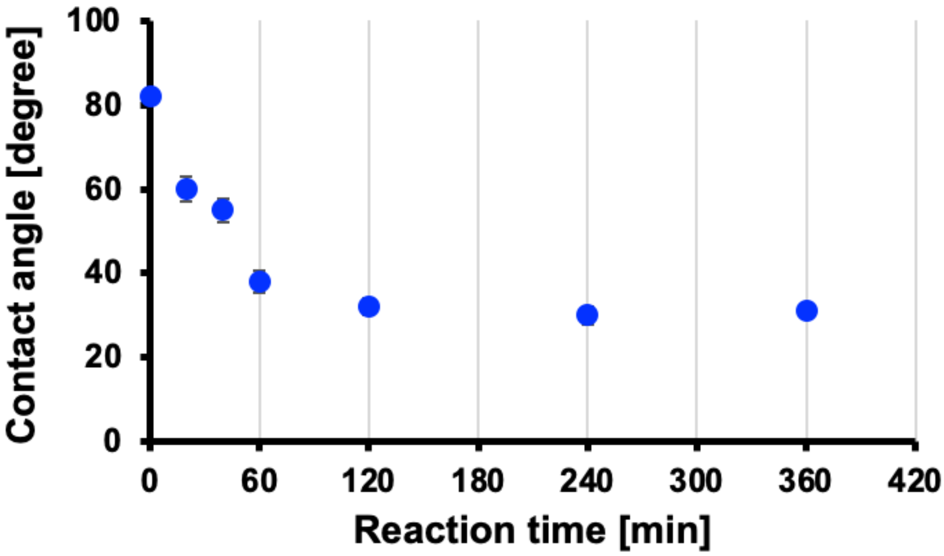

Figure S6. Relationship between reaction time and contact angle when reacting with 2.5 M NaOH at 50°C. The contact angle became the smallest at a reaction time of 120 minutes, after which there was almost no change. Therefore, the reaction time was set to 120 minutes.

3. Effect of the Film Gap on Radial Velocities

Finite Element Analysis was conducted to investigate the effect of the film interval of the 3D-Stack on the flow velocity. COMSOL Multiphysics ver.5.6 was used as the analysis software. A 2D axial symmetry model was used as the analytical model (Figure S7). The flow is assumed to be laminar, and the incompressible Navier-Stokes equation (1) and Continuous equation (2) were calculated. As the boundary condition, a no-slip condition was applied to the well wall and the 3D-Stack surface, and a symmetric boundary was applied to the gas-liquid interface due to the small deformation of the free surface. A pressure constraint is applied to the corner of the well to solve NS equation. The material properties of the solution were given as the value of water at 20°C (Kinematic viscosity : 1.004 cSt, density : 998 kg/m^3).

$$\frac{\partial \boldsymbol{v}}{\partial t} + (\boldsymbol{v} \cdot \nabla)\boldsymbol{v} = -\frac{1}{\rho}\operatorname{grad} p + \nu \triangle \boldsymbol{v} + \boldsymbol{g} \quad (1)$$

$$\frac{\partial \rho}{\partial t} + \nabla \cdot (\rho \boldsymbol{v}) = 0 \quad (2)$$

Nomenclature

$\boldsymbol{v}$	velocity vector [m/s]	ρ	density [kg・m^3]
$\boldsymbol{g}$	pressure [Pa]	p	pressure [Pa]
ν	kinematic viscosity [m^2/s]	t	time [s]

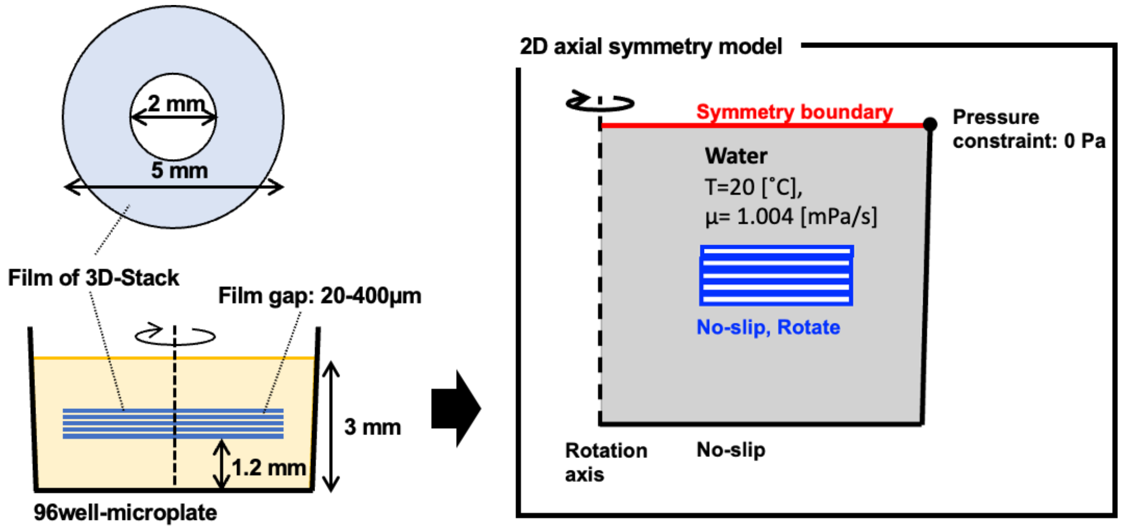

Figure S7. Analytical model.

The radial velocity at the outlet is shown in Figure S8. The larger the film gap, the smaller the radial flow velocity. Therefore, increasing the film gap could reduce the circulation rate of the solution and decrease the reaction efficiency.

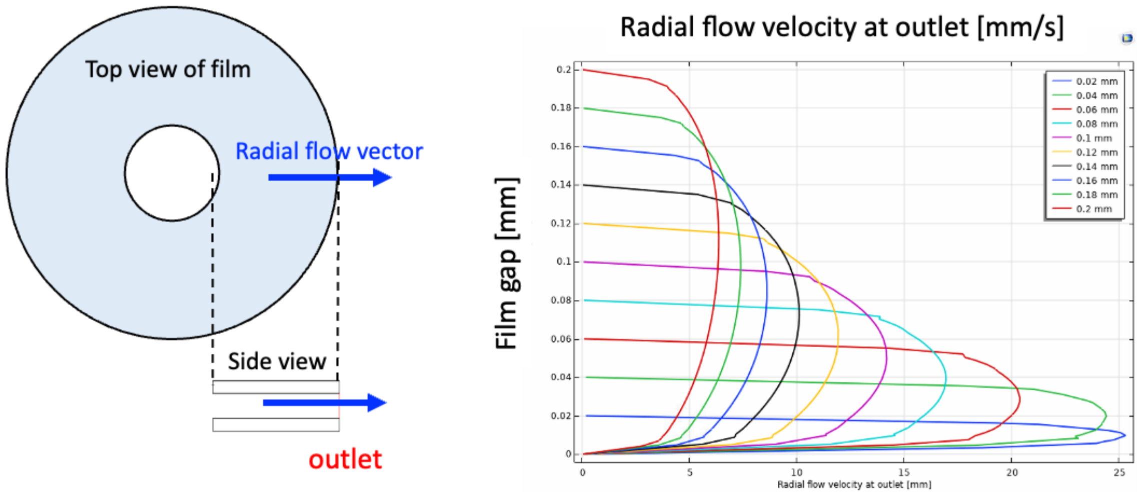

Figure S8. Effect of film gap on radial velocity.

Reference

1. Awaja, F. and D. Pavel, Recycling of PET. *Eur. Poly. J.* **2005**. *41*, 1453–1477.
2. Atthoff, B. and J. Hilborn, Protein adsorption onto polyester surfaces: Is there a need for surface activation? *J. Biomed. Mater. Res. Part B-Appl. Biomater.* **2007**, **80B**, 21–130.
